# Supplementary figures and images for: Assessing multiple score functions in Rosetta for drug discovery
Source: PLoS One. 2020 Oct 12;15(10):e0240450. doi: 10.1371/journal.pone.0240450 (PMC7549810; doi:10.1371/journal.pone.0240450)

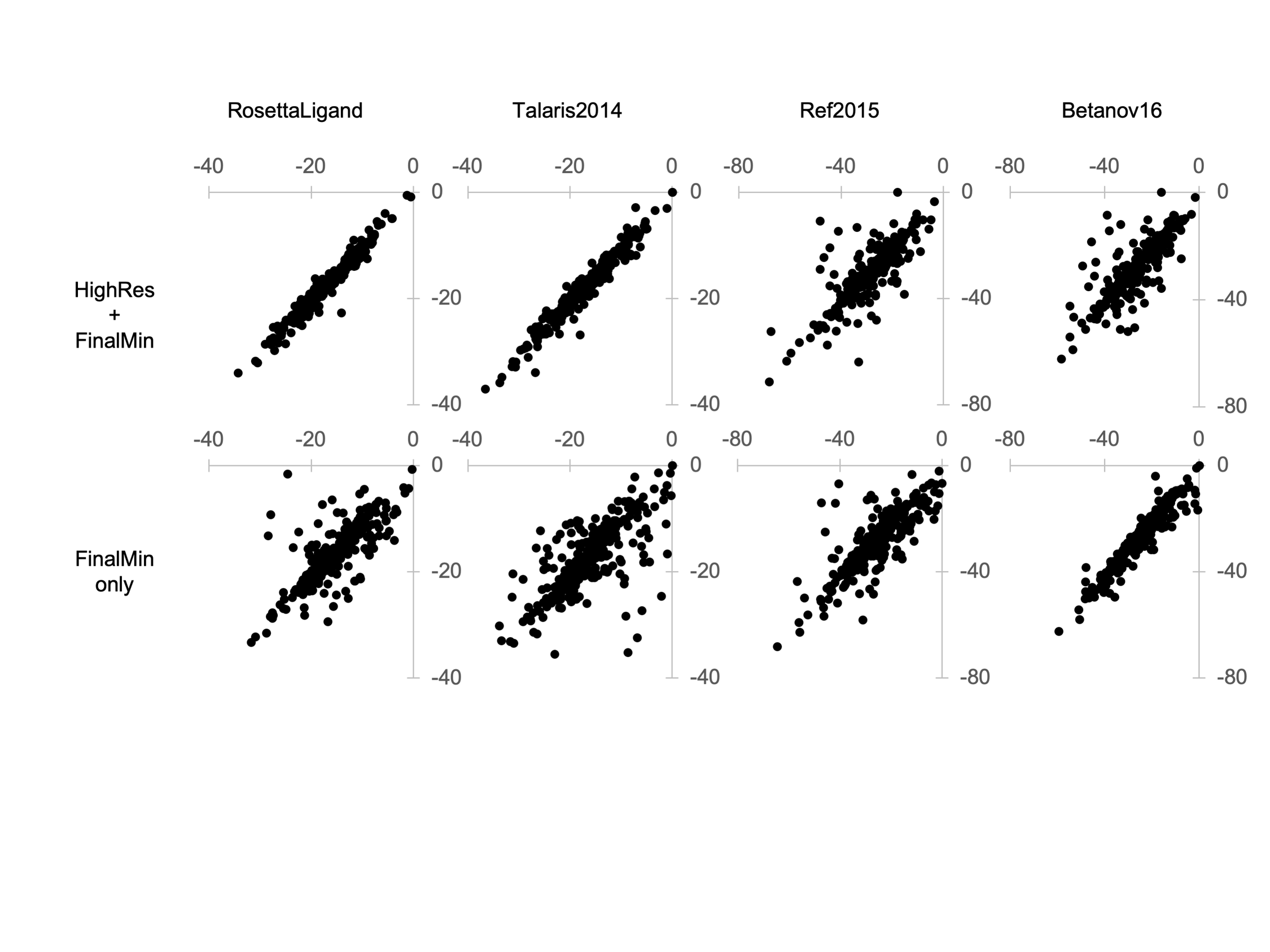

Supplement: S1 Fig — Scores from raw and opt set are depicted on the x and y axes, respectively. (TIF) [file pone.0240450.s001.tif]

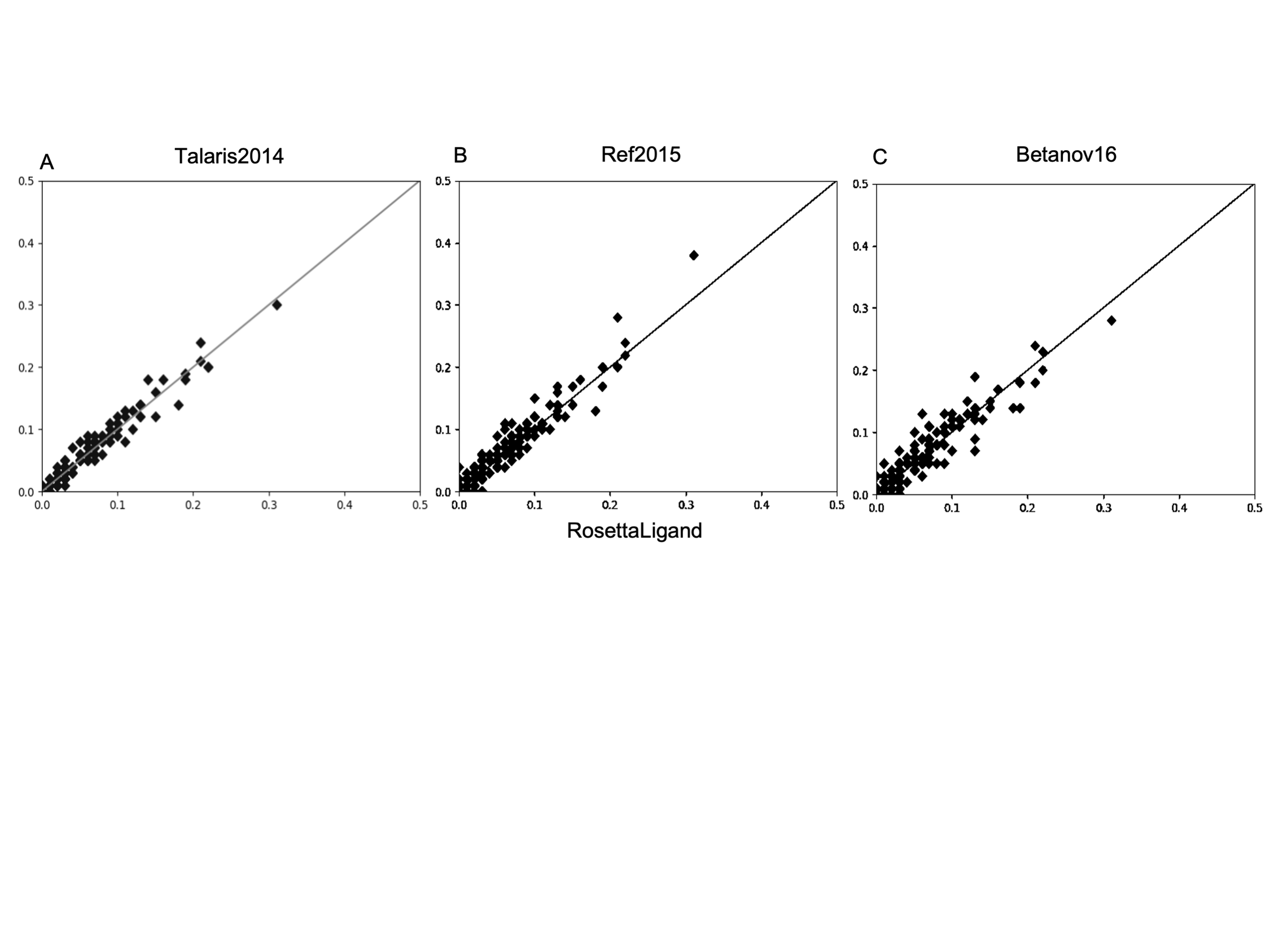

Supplement: S2 Fig — RosettaLigand value on the x-axis and Talaris2014 (A), Ref2015 (B) and Betanov16 (C) on the y-axis. (TIF) [file pone.0240450.s002.tif]

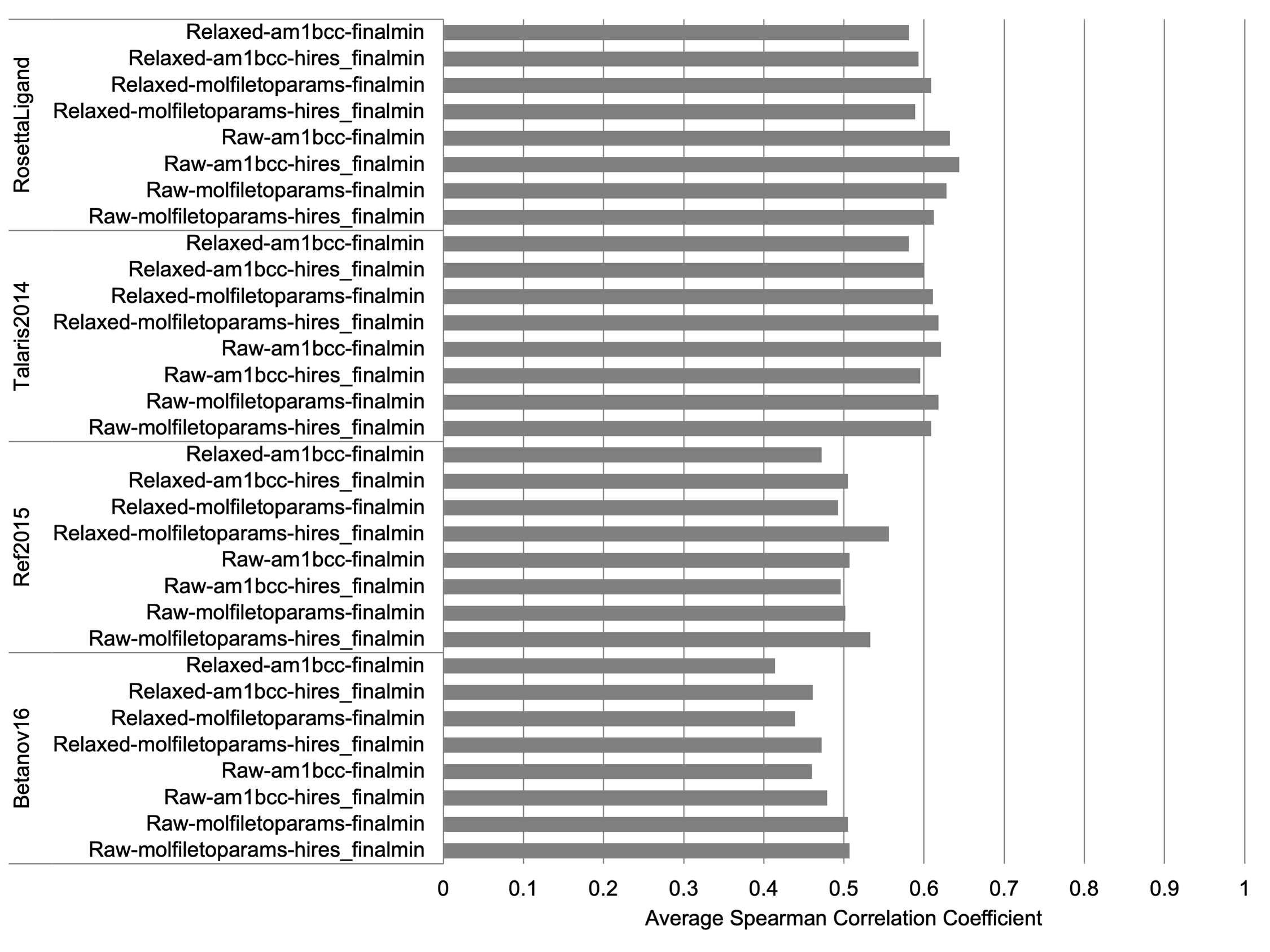

Supplement: S3 Fig — Average Spearman correlation across multiple preparation protocols: un-optimized PDB file “raw” and constrained relax “relaxed” protein preparations, molfiletoparams.py script “molfiletoparams” and AM1-BCC “am1bcc” partial charge generation, and high-resolution sampling and final minimization “hires_finalmin” vs. only final minimization “finalmin”. Each combination was tested within each score function. (TIF) [file pone.0240450.s003.tif]
