# Supplementary material for: Assessing multiple score functions in Rosetta for drug discovery
Source: PLoS One. 2020 Oct 12;15(10):e0240450. doi: 10.1371/journal.pone.0240450 (PMC7549810; doi:10.1371/journal.pone.0240450)
Supplement: S1 File — (DOCX) [file pone.0240450.s004.docx]

Protocol Capture:

This protocol capture contains all preparation, run and analysis scripts for CASF-2016 tests. Additionally, we also provide the setup for docking with full sampling test and AM1-BCC partial charge generation method. All CASF-2016 experiments use the inputs and analysis scripts provided by the CASF-2016 distribution described in (1) The dataset including all input files and analysis scripts was downloaded from <http://www.pdbbind-cn.org/casf.php> . All Rosetta scripts were run using version 3.10.

Some scripts below using OpenBabel and BCL software from the following publications.

1. Su M.Y.; Yang Q.F.; Du Y.; Feng G.Q.; Liu Z.H.; Li Y.; Wang R.X. Comparative Assessment of Scoring Functions: The CASF-2016 Update. *J. Chem. Inf. Model.*, **2019**. DOI: 10.1021/acs.jcim.8b00545.
2. O'Boyle NM, Banck M, James CA, Morley C, Vandermeersch T, Hutchison GR. Open Babel: An open chemical toolbox. J Cheminform. 2011;3:33.
3. Kothiwale S, Mendenhall JL, Meiler J. BCL::Conf: small molecule conformational sampling using a knowledge based rotamer library. J Cheminform. 2015;7:47.
4. Jakalian A, Jack DB, Bayly CI. Fast, efficient generation of high-quality atomic charges. AM1-BCC model: II. Parameterization and validation. J Comput Chem. 2002;23(16):1623-41.

# CASF-2016 Tests:

## Preparation of ligand files for scoring/ranking tests:

## Takes ligand compound files given from CASF2016, labelled as "raw" and "opt" to generate Rosetta-readable files. Each ligand was put into its own subdirectory ${PDB}/ based on the PDB ID (4-letter code).

PDB=$1 ## 4-letter PDB code

SFXN=$2 ## score function

RUN=$3 ## using ”raw” or “opt” structure

# Use Open Babel (2.4.1) to convert .mol2 to .sdf format

obabel -imol2 ${PDB}/${PDB}_ligand_${TYPE}.mol2 -osdf -O ${PDB}/${PDB}_ligand_${TYPE}.sdf

# Generate Rosetta-readable params file for ligand. This outputs a .pdb file containing matched atom names to the params file and a ligand parameters file (.params).

python2.7 $ROSETTA/main/source/scripts/python/public/molfile_to_params.py -n ${PDB}_ligand_${TYPE} -p ${PDB}_ligand_${TYPE} --mm-as-virt --long-names --chain X ${PDB}/${PDB}_ligand_${TYPE}.sdf

# Move outputs from molfiletoparams.py script into appropriate subdirectory.

mv ${PDB}*.p* ${PDB}/

    done

done

## Running scoring/ranking tests:

SFXN=$1 ## score function

RUN=$2 ## using ”raw” or “opt” structure

TYPE=$3 ## hires + finalmin or just finalmin

$ROSETTA/main/source/bin/rosetta_scripts.linuxgccrelease \

    @ ${TYPE}.flags \

    @ ${SFXN}.flags \

    -parser:protocol ${SFXN}_${TYPE}.xml \

    -s "$CASF/coreset/${PDB}/${PDB}_protein.pdb \ $CASF/coreset/${PDB}/${PDB}_ligand_raw_0001.pdb" \

    -in:file:native "$CASF/coreset/${PDB}/${PDB}_protein.pdb \ $CASF/coreset/${PDB}/${PDB}_ligand_raw_0001.pdb" \

    -extra_res_fa $CASF/coreset/${PDB}/${PDB}_ligand_raw.params \

    -out:file:score_only ${PDB}_score.sc \

Running scoring and ranking test analysis scripts:

# Following file formatting in CASF examples, .dat files require “#code score” in first line followed by PDBID and score on the following rows.

# Scoring test analysis

python2.7 $CASF/power_scoring/scoring_power.py -c $CASF/power_scoring/CoreSet.dat -s run.dat -p 'negative' -o run_scoringtest > run_scoringtest.out

# Ranking test analysis

python2.7 $CASF/power_ranking/ranking_power.py -c $CASF/power_scoring/CoreSet.dat -s run.dat -p 'negative' -o run_rankingtest > run_rankingtest.out

# Subset analysis

for SUBSET in H S V ; do for NUM in {1..3}; do

python2.7 $CASF/power_scoring/scoring_power.py -c $CASF/power_scoring/subset-${SUBSET}${NUM}.dat -s run.dat -p 'negative' -o run_scoringtest.${SUBSET}${NUM} > run_scoringtest.${SUBSET}${NUM}.out

python2.7 $CASF/power_ranking/ranking_power.py -c $CASF/power_scoring/subset-${SUBSET}${NUM}.dat -s run.dat -p 'negative' -o run_rankingtest.${SUBSET}${NUM} > run_rankingtest.${SUBSET}${NUM}.out

done done

Preparation of ligand files for docking test:

# Start from ${PDB}_decoys.mol2 file distributed from CASF2016 and split into individual decoy .mol2 files into PDB_DECOYNUMBER_raw.mol2.

# Similar to ligand preparation for the scoring/ranking test, we have to make these Rosetta-readable ligand files.

# listof_ligands text file contains the 4-letter PDB ID on each line

for LIGAND in `awk '{print $1}' ./listof_ligands `; do

obabel -imol2 ${LIGAND}_raw.mol2 -osdf -O ${LIGAND}_clean.sdf

python2.7 $ROSETTA/main/source/scripts/python/public/molfile_to_params.py -n ${LIGAND} -p ${LIGAND} --mm-as-virt --conformers-in-one-file ${LIGAND}_clean.sdf --chain X

done

Running docking test:

# Start from ${PDB}_decoys.mol2 file distributed from CASF2016 and split into individual decoy .mol2 files into PDB_DECOYNUMBER_raw.mol2.

# Similar to ligand preparation for the scoring/ranking test, we have to make these Rosetta-readable ligand files.

# listof_ligands text file contains the 4-letter PDB ID on each line

for LIGAND in `awk '{print $1}' ./listof_ligands `; do

obabel -imol2 ${LIGAND}_raw.mol2 -osdf -O ${LIGAND}_clean.sdf

python2.7 $ROSETTA/main/source/scripts/python/public/molfile_to_params.py -n ${LIGAND} -p ${LIGAND} --mm-as-virt --conformers-in-one-file ${LIGAND}_clean.sdf --chain X

done

Running docking test analysis scripts:

Running docking test analysis:

# Following file formatting in CASF examples, all PDBs are put into a single directory (e.g. ./docking_run) with filenames $PDB_score.dat. require “#code score” in first line followed by decoy name and score on the following rows.

# Docking test analysis

python2.7 $CASF/power_docking/docking_power.py -c $CASF/power_docking/CoreSet.dat -s ./docking_run/ -p 'negative' -r $CASF/decoys_docking/ -o run_dockingtest > run_dockingtest.out

# Subset analysis

for SUBSET in H S V ; do for NUM in {1..3}; do

python2.7 $CASF/power_docking/docking_power.py -c $CASF/power_docking/subset-${SUBSET}${NUM}.dat -s ./docking_run/ -p 'negative' -r $CASF/decoys_docking/ -o run_dockingtest.${SUBSET}${NUM} > run_dockingtest.${SUBSET}${NUM}.out

done done

Preparation of ligand file for screening test:

# convert given .mol2 file containing decoys into individual SDFs.

obabel -imol2 ${PDB}_${LIG}.mol2 -osdf -O ${PDB}_ligand_${LIG}.sdf

python ./split_sdffile.py ${PDB}_ligand_${LIG}.sdf

for i in ${LIG}_ligand_*sdf ; do

python2.7 $ROSETTA/main/source/scripts/python/public/molfile_to_params.py -n LIG -p ${i%.sdf} --long-names --mm-as-virt --chain X ${i}

done

ls *.pdb >> listof_${PDB}_${LIG}.pdbs

## Running screening test

$ROSETTA/main/source/bin/rosetta_scripts.linuxgccrelease \

| @ flags_common_nstruct1 \      @ flags_${SFXN} \      -parser:protocol ${SFXN}_finalmin.xml \      -s ${PDB}_protein.pdb ${PDB}_ligand_${LIG}.pdb" \      -in:file:native ${PDB}_protein.pdb ${PDB}_ligand_${LIG}.pdb" \      -extra_res_fa ${PDB}_ligand_${LIG}.params \      -out:file:scorefile ./screening_run/${PDB}_${LIG}.sc |
| --- |

## Running screening test analysis:

# Following file formatting in CASF examples, all PDBs are put into a single directory (e.g. ./screening_run) with filenames $PDB_score.dat. require “#code score” in first line followed by decoy name and score on the following rows.

# Screening test analysis

python2.7 $CASF/power_screening/screening_power.py -c $CASF/power_screening/CoreSet.dat -s ./screening_run/ -p 'negative' -r $CASF/decoys_screening/ -o run_screeningtest > run_screeningtest.out

# Subset analysis

for SUBSET in H S V ; do for NUM in {1..3}; do

python2.7 $CASF/power_screening/screening_power.py -c $CASF/power_screening/subset-${SUBSET}${NUM}.dat -s ./screening_run/ -p 'negative' -r $CASF/decoys_screening/ -o run_screeningtest.${SUBSET}${NUM} > run_screeningtest.${SUBSET}${NUM}.out

done done

# Docking test with full sampling

## Preparation of ligand files

## AM1-BCC partial charge generation:

# separate into directory by PDB ID.

mkdir -p ./ligand_prep_for_docking/${PDB}/

cd ./ligand_prep_for_docking/${PDB}/

# make 2d structure in obabel so we don't start from docked structure

obabel -imol $coreset/${PDB}/${PDB}_ligand_raw.sdf -osdf -O ${PDB}_2d.sdf --gen2d

# make idealized 3d from 2d

obabel -isdf ${PDB}_2d.sdf -osdf -O ${PDB}_3d.sdf --gen3d

# check in bcl

bcl.exe molecule:Filter -add_h -neutralize -defined_atom_types -3d -input_filenames ${PDB}_3d.sdf -output_matched ${PDB}_CLEANED.sdf -output_unmatched ${PDB}_UNCLEANED.sdf -message_level Debug

# generate conformers

bcl.exe molecule:ConformerGenerator -rotamer_library cod -top_models 100 -ensemble_filenames ${PDB}_CLEANED.sdf -conformers_single_file ${PDB}_CLEANED.conf.sdf -conformation_comparer 'Dihedral(method=Max)' 30 -max_iterations 1000

# generate params file

python2.7 /dors/meilerlab/apps/rosetta/rosetta-3.10/main/source/scripts/python/public/molfile_to_params.py -n ${PDB}_ligand -p ${PDB}_ligand --mm-as-virt --long-names --conformers-in-one-file ${PDB}_CLEANED.conf.sdf --chain X

# AM1-BCC Partial Charge Generation:

/programs/x86_64-linux/ambertools/17/bin/antechamber -i $coreset_dir/${PDB}/${PDB}_ligand_opt.sdf -fi sdf -o $coreset_dir/${PDB}/${PDB}_am1bcc.mol2 -fo mol2 -c bcc -nc ${FC}

XMLs and options files for Rosetta runs:

Basic XML:

<ROSETTASCRIPTS>

    <SCOREFXNS>

        [**SCORE FUNCTION CHANGES HERE**]

    </SCOREFXNS>

    <TASKOPERATIONS>

    </TASKOPERATIONS>

    <LIGAND_AREAS>

        <LigandArea name="docking_sidechain" chain="X" cutoff="6.0" add_nbr_radius="true" all_atom_mode="true" minimize_ligand="10"/>

        <LigandArea name="final_sidechain" chain="X" cutoff="6.0" add_nbr_radius="true" all_atom_mode="true"/>

        <LigandArea name="final_backbone" chain="X" cutoff="7.0" add_nbr_radius="false" all_atom_mode="true" Calpha_restraints="0.3"/>

    </LIGAND_AREAS>

    <INTERFACE_BUILDERS>

        <InterfaceBuilder name="side_chain_for_docking" ligand_areas="docking_sidechain"/>

        <InterfaceBuilder name="side_chain_for_final" ligand_areas="final_sidechain"/>

        <InterfaceBuilder name="backbone" ligand_areas="final_backbone" extension_window="3"/>

    </INTERFACE_BUILDERS>

    <MOVEMAP_BUILDERS>

        <MoveMapBuilder name="docking" sc_interface="side_chain_for_docking" minimize_water="true"/>

        <MoveMapBuilder name="final" sc_interface="side_chain_for_final" bb_interface="backbone" minimize_water="true"/>

    </MOVEMAP_BUILDERS>

    <SCORINGGRIDS ligand_chain="X" width="30.0">

        <ClassicGrid grid_name="vdw" weight="1.0"/>

    </SCORINGGRIDS>

    <MOVERS>

**[DELETE LEADING “<” TO REMOVE HighResDocker stage on line below]**

<HighResDocker name="high_res_docker" cycles="6" repack_every_Nth="3" scorefxn="ligand_soft_rep" movemap_builder="docking"/>

        <FinalMinimizer name="final" scorefxn="hard_rep" movemap_builder="final"/>

        <InterfaceScoreCalculator name="add_scores" chains="X" scorefxn="hard_rep" compute_grid_scores="0"/>

**[DELETE LEADING “<” TO REMOVE HighResDocker stage on line below]**

        <ParsedProtocol name="high_res_dock">

            <Add mover_name="final"/>

        </ParsedProtocol>

        <ParsedProtocol name="reporting">

            <Add mover_name="add_scores"/>

        </ParsedProtocol>

    </MOVERS>

    <PROTOCOLS>

        <Add mover_name="high_res_dock"/>

        <Add mover_name="reporting"/>

    </PROTOCOLS>

</ROSETTASCRIPTS>

Adaptations to above XML for respective score functions:

RosettaLigand

<ScoreFunction name="ligand_soft_rep" weights="ligand_soft_rep">

     <Reweight scoretype="fa_elec" weight="0.42"/>

     <Reweight scoretype="hbond_bb_sc" weight="1.3"/>

     <Reweight scoretype="hbond_sc" weight="1.3"/>

     <Reweight scoretype="rama" weight="0.2"/>

</ScoreFunction>

<ScoreFunction name="hard_rep" weights="ligand">

     <Reweight scoretype="fa_intra_rep" weight="0.004"/>

     <Reweight scoretype="fa_elec" weight="0.42"/>

     <Reweight scoretype="hbond_bb_sc" weight="1.3"/>

     <Reweight scoretype="hbond_sc" weight="1.3"/>

     <Reweight scoretype="rama" weight="0.2"/>

</ScoreFunction>

Talaris2014

<ScoreFunction name="ligand_soft_rep" weights="ligand_soft_rep">

     <Reweight scoretype="fa_elec" weight="0.42"/>

     <Reweight scoretype="hbond_bb_sc" weight="1.3"/>

     <Reweight scoretype="hbond_sc" weight="1.3"/>

     <Reweight scoretype="rama" weight="0.2"/>

</ScoreFunction>

<ScoreFunction name="hard_rep" weights="talaris2014_cst">

</ScoreFunction>

Ref2015

<ScoreFunction name="ligand_soft_rep" weights="beta_nov15_soft">

<Reweight scoretype=”coordinate_constraint” weight=”1.0”/>

<Reweight scoretype=”atom_pair_constraint” weight=”1.0”/>

<Reweight scoretype=”angle_constraint” weight=”1.0”/>

<Reweight scoretype=”dihedral_constraint” weight=”1.0”/>

<Reweight scoretype=”chainbreak” weight=”1.0”/>

<ScoreFunction>

<ScoreFunction name=”hard_rep” weights=”beta_nov15_cst”>

</ScoreFunction>

Betanov16

<ScoreFunction name="ligand_soft_rep" weights="beta_nov15_soft">

<Reweight scoretype=”coordinate_constraint” weight=”1.0”/>

<Reweight scoretype=”atom_pair_constraint” weight=”1.0”/>

<Reweight scoretype=”angle_constraint” weight=”1.0”/>

<Reweight scoretype=”dihedral_constraint” weight=”1.0”/>

<Reweight scoretype=”chainbreak” weight=”1.0”/>

<ScoreFunction>

<ScoreFunction name=”hard_rep” weights=”beta_nov15_cst”>

</ScoreFunction>

Flags files for respective score functions using Rosetta v3.10: (e.g. ${SFXN}.flags)

These options turn are used to ensure compatibility with database and scoring files when using a particular score function. The Ref2015 flags file is empty; however, since I ran these within a loop, the file did need to exist in order to not crash out due to a file not existing.

RosettaLigand

-mistakes:restore_pre_talaris_2013_behavior

-store:analytic_etable_evaluation true

Talaris2014

-restore_talaris_behavior true

Ref2015

# Not needed since Ref2015 is currently the default score function in Rosetta v3.10.

Betanov16

-beta_nov16
