# Supplementary material for: Assessing multiple score functions in Rosetta for drug discovery
Source: PLoS One. 2020 Oct 12;15(10):e0240450. doi: 10.1371/journal.pone.0240450 (PMC7549810; doi:10.1371/journal.pone.0240450)
Supplement: Attachment — Submitted filename: This is a great paper.docx [file pone.0240450.s005.docx]

This is a great paper, that describes careful testing of a state of the art code, new developments in scoring functions for small-molecule to macromolecule interaction modeling and docking.

The paper provides a important negative result, that recent developments have actually decreased performance in spite of reports from the various groups to the contrary. I am inclined to think that this paper is an important part of a discussion about reproducibility and progress in this field.

Although I am strongly in favor of publication, I would point out that the paper needs editing for clarity and writing style.

Should you remove the subheadings from the introduction?

The introduction needs to be increased in depth and completeness. In particular the last section of the introduction needs to be improved.

 The results section needs a preamble to set up the discussion there. Currently it just jumps into a detail and could lead to confusion.

Define “subsets” better and earlier.

Defining “scoring power” is unnecessary, it is just the Rho to the true affinity, so just call it that. In fact, the word “power” could be deleted everywhere in the manuscript and the result would be an increase in clarity.

Subheading “We also test across a range of preparation…” should be “We test across a range of preparation…”
