# Supplementary material for: Assessing multiple score functions in Rosetta for drug discovery
Source: PLoS One. 2020 Oct 12;15(10):e0240450. doi: 10.1371/journal.pone.0240450 (PMC7549810; doi:10.1371/journal.pone.0240450)
Supplement: Attachment — Submitted filename: Response to Reviewers.docx [file pone.0240450.s006.docx]

Dear Emilio Gallicchio,

We have carefully analyzed the reviewer comments and revised our manuscript entitled *Assessing multiple score functions in Rosetta for Drug Discovery* following their suggestions. We feel that the manuscript substantially improved. We hope that you will find this revised version acceptable for publication in *Plos One*. Please find a detailed summary of all changes applied to the manuscript and our point-by-point responses to the reviewer comments below.

Best regards,

Shannon Smith

**Response to Reviewer #1**

We are appreciative of the Reviewer for providing comments and suggestions to further improve this manuscript. The primary recommendation from the Reviewer is to discuss biophysical differences between scorefunctions. The Reviewer also recommends clarity in describing results, specifically comparing Rosetta protocols to other software in Figure 2. Additional comments address the differentiation of “activity” and “binding” terms. In the revised version, we have addressed each of these points as described below.

1. **The Reviewer commented:**

*The study outlines the assessment of several Rosetta energy functions for the RosettaLigand method. This study was necessary since the recent versions of the score functions show reduced performance across small molecule benchmarks and an appropriate dataset from CASF-2016 was selected to test this. Overall, the manuscript covers this topic in depth and with the expected detail in examination, my recommendation is to accept with revisions.*

Thank you for your input to improve this manuscript, as well as supporting the necessity of this study.

2. **The Reviewer commented:**

*The authors mention activity and binding as interchangeable terms. Rosetta or any other platform is unlikely to measure activity per se and energies measured are a more direct measure of binding. Appropriate correction/ discussion of this topic should be included to avoid misuse of this method.*

The reviewer raises an excellent point, as these methods aim to provide insight on binding, not necessarily activity. To clarify this point, the authors have substituted each instance of “active” for “binder” throughout the text.

“actives” to “binders” (Line 71)

“actives” to “binders” and “non-actives” to “non-binders” (Line 182)

“activity” to “binding” (Line 288)

3. **The Reviewer commented:**

*Fig 2 - this figure does not specifically demonstrate how rosetta ranks 2/34 out of tested methods. Either a better graphical representation needs to be chosen or a better wording to explain this. The top 4 score functions seem to show similar values and error especially in graph B. Authors would need to further elaborate this point better.*

Thank you for raising this point. We have added the following sentence to the Figure 2 caption.

“For simplicity, only the top performing score function from each external program is presented here.”

The following sentence from the Ranking Test Results section has also been revised to include performance similarity to the other programs. (line 250-251).

“In the ranking test, we obtain an average Spearman correlation of 0.63 using the RosettaLigand score function, which performs similarly to DrugScoreCSD, ChemPLP@GOLD and LUDI2@DS.”

4. **The Reviewer commented:**
   *In general, it would be good to discuss the biophysical significance of each score function chosen perhaps in describing how they score the same ligand bound target.*

We appreciate this comment, as we acknowledge that this is an important point to include. To address this recommendation, the revision now includes a new paragraph in the Introduction section to briefly describe the biophysical significance and basis of Rosetta score functions.

“While the terms of the Rosetta score functions are similar to terms used in molecular mechanics, the shape of the potential is mostly determined from statistics over geometries observed in experimentally determined structures deposited in the Protein Data Bank (PDB) ([www.rcsb.org](http://www.rcsb.org)) [22]. It is assumed that structural features commonly observed in the PDB knowledge base are energetically more favorable. By using a Boltzmann equation, this probability is converted into a pseudo energy. Although these score terms are largely inspired by statistics, the corresponding weights have been optimized to some extent to align with experimentally observed free energies. Here, we provide a brief overview of score functions throughout Rosetta development; a comprehensive score function overview is presented in [23, 24].”

(lines 81-89)

5. **The Reviewer commented:**

*Typographical error: Line 59 these calculations are “a” resource intensive approach*

Thank you for catching this and it has been edited accordingly.

**Response to Reviewer #2**

We are appreciative of the Reviewer for providing comments and suggestions to further improve this manuscript. The primary recommendations of the Reviewer are to improve writing clarity, specifically by expanding the introduction to be more complete and improving the transition into the Results section. Additional comments recommend clarity in terminology, specifically when introducing “subsets” and using “power” in the names of the different tests. In the revised version, we have addressed each of these points as described below.

1. **The Reviewer commented:**

*This is a great paper, that describes careful testing of a state of the art code, new developments in scoring functions for small-molecule to macromolecule interaction modeling and docking.*

*The paper provides a important negative result, that recent developments have actually decreased performance in spite of reports from the various groups to the contrary. I am inclined to think that this paper is an important part of a discussion about reproducibility and progress in this field. Although I am strongly in favor of publication, I would point out that the paper needs editing for clarity and writing style.*

We greatly appreciate the Reviewers support for publication and valuable feedback for improving the caliber of the manuscript. We are also appreciative for the Reviewer’s comments regarding the importance of reproducibility in this field.

2. **The Reviewer commented:**

*Should you remove the subheadings from the introduction?*

The subheadings have been converted to be the first sentence of each section without any change in wording except for one instance for grammatical reasons (line 79).

“Here, we provide a brief overview of score functions throughout Rosetta development.”

3. **The Reviewer commented:**

*The introduction needs to be increased in depth and completeness. In particular the last section of the introduction needs to be improved.*

The Reviewer requests a more in-depth description in the introduction, specifically in the last section regarding the ligand docking with full sampling test. Following the Reviewer’s recommendation, we have added several sentences to further explain why we decided to add this test along with the CASF-2016 tests. (lines 118-130)

“In addition to CASF-2016 tests, we also perform ligand docking with full sampling to investigate performance differences between Rosetta score functions in a more realistic test-case for users. Ligand docking with full sampling uses the standard Rosetta protocol which allows extensive flexible binding pocket sampling to obtain diverse ligand poses while using a score function to correctly identify the native binding mode. The purpose of this test is to determine how well each score function favorable scores the native binding mode against non-native decoy poses. Importantly, using poses sampled by Rosetta instead of pre-selected binding modes by CASF-2016 allows us to extract specific protein-ligand interactions considered favorable or unfavorable in each score function. Using these insights along with results from the other diverse CASF-2016 tests, this provides a more thorough benchmark to guide future work.”

4. **The Reviewer commented:**

*The results section needs a preamble to set up the discussion there. Currently it just jumps into a detail and could lead to confusion.*

We appreciate the Reviewer’s recommendation and agree that this needs a better transition. We have added the following title and first two sentences at the beginning of the Results section to improve this. (lines 132-138)

## “Starting structures for each benchmark are provided by CASF-2016 datasets.

CASF-2016 provides all structural input data for each benchmark test in aims to reduce sampling biases between score functions and allowing for direct comparison between methods. In the scoring and ranking tests, which use a known structure to correlate score with experimental binding, CASF-2016 provides “raw” structures directly from the Protein Data Bank ([www.rcsb.org](http://www.rcsb.org))(30) without any optimization or structure modification.”

5. **The Reviewer commented:**

*Define “subsets” better and earlier.*

Thank you for this recommendation. We have moved the subset definition up to the second paragraph of the Results section and expanded the explanation briefly to better explain the purpose. (Lines 162-170)

“For each benchmark test, we present protein subset results according to the CASF-2016 defined criteria to understand the effects of the binding pocket environment on performance, specifically the size, solvent exposure and hydrophobicity of surrounding residues.”

6. **The Reviewer commented:**

*Defining “scoring power” is unnecessary, it is just the Rho to the true affinity, so just call it that. In fact, the word “power” could be deleted everywhere in the manuscript and the result would be an increase in clarity.*

We greatly appreciate the Reviewer’s comment on this to improve the clarity of the manuscript. In response, the term “power” has been replaced accordingly throughout the manuscript where e.g. “scoring power test” is now “scoring test”. Additionally, the introduction to each test now uses the appropriate statistical term to define what is being calculated.

“The scoring test uses the Pearson correlation to analyze a score function’s ability to linearly correlate the predicted interaction with experimental binding affinity across the full 285 protein-ligand complex dataset.” (lines 210)

“The ranking test uses the Spearman correlation to analyze a score function’s ability to rank binding affinities of compounds against the same protein target.” (line 236)

7. **The Reviewer commented:**

*Subheading “We also test across a range of preparation…” should be “We test across a range of preparation…”*

This has been changed according to the recommendation.
